# 

**Published:** 1874-03

**Authors:** 


VOLUME XI. | MARCH—1874. | NUMBER 12.

### ATLANTA
### Medical and Surgical Journal.

MONTHLY: THREE DOLLARS PER ANNUM, IN ADVANCE.

ROBERT BATTEY, M.D.,

EDITOR.

WM. ABRAM LOVE, M.D., V. H. TALIAFERRO, M.D.,
ASSOCIATE EDITORS.

*PAX ET SCIENTIA, SED VERITAS SINE TIMORE.*

ATLANTA, GEORGIA:
*W. R. BARROW, BOOK AND GENERAL JOB PRINTER.*
1874.

